# Cidea Targeting Protects Cochlear Hair Cells and Hearing Function From Drug‐ and Noise‐Induced Damage

**DOI:** 10.1002/advs.202517206

**Published:** 2025-11-20

**Authors:** Shasha Zhang, Ruiying Qiang, Yuan Zhang, Jinxian Wan, Chen Tao, Ying Dong, Xujun Tang, Li Xu, Hairong Xiao, Yanqin Lin, Wei Tong, Ying Ma, Yongming Wang, Peng Li, Renjie Chai

**Affiliations:** ^1^ State Key Laboratory of Digital Medical Engineering Department of Otolaryngology Head and Neck Surgery Zhongda Hospital School of Life Sciences and Technology Advanced Institute for Life and Health Jiangsu Province High‐Tech Key Laboratory for Bio‐Medical Research Southeast University Nanjing 210096 China; ^2^ Southeast University Shenzhen Research Institute Shenzhen 518063 China; ^3^ Department of Otolaryngology Head and Neck Surgery Sichuan Provincial People's Hospital University of Electronic Science and Technology of China Chengdu 610072 China; ^4^ Co‐Innovation Center of Neuroregeneration Nantong University Nantong 226001 China; ^5^ Institute for Stem Cell and Regeneration Chinese Academy of Science Beijing 100101 China; ^6^ Tsinghua‐Peking Center for Life Sciences School of Life Sciences Tsinghua University Beijing 100084 China; ^7^ State Key Laboratory of Metabolic Dysregulation & Prevention and Treatment of Esophageal Cancer; Tianjian Laboratory of Advanced Biomedical Sciences Academy of Medical Sciences Zhengzhou University Zhengzhou 450001 China; ^8^ Center for Medical Research and Innovation Shanghai Pudong Hospital Fudan University Pudong Medical Center, Shanghai Engineering Research Center of Industrial Microorganisms Fudan University Shanghai 200438 China; ^9^ Department of Otolaryngology‐Head and Neck Surgery Nanjing Drum Tower Hospital Affiliated Hospital of Medical School Nanjing University Nanjing 210008 China; ^10^ Research Institute of Otolaryngology Nanjing 210061 China

**Keywords:** Cidea, gene editing, neomycin, noise, sensorineural hearing loss

## Abstract

Acquired sensorineural hearing loss (SNHL) is primarily caused by the damage or loss of hair cells (HCs), induced by factors such as noise exposure and ototoxic drugs. However, clinical treatments for SNHL remain limited. Here, the role of the apoptosis‐inducing gene ***Cidea*** in SNHL is investigated. It is initially observed that ***Cidea*** expression is specifically increased in neomycin‐damaged HCs at both the protein and mRNA levels. To further explore its role, ***Cidea*** knockout (Cidea‐/‐) mice are obtained, and it is found that the absence of ***Cidea*** effectively alleviates HC apoptosis caused by neomycin treatment and noise exposure in vivo. Moreover, a novel therapeutic strategy for SNHL has been developed by delivering ***CRISPR/SlugCas9‐HF*** via AAV to edit ***Cidea***, and this approach significantly reduced HC loss induced by both neomycin and noise exposure. These findings suggest that ***Cidea*** may serve as a promising target for the prevention of neomycin‐ and noise‐induced SNHL in clinical settings.

## Graphical abstract

Acquired sensorineural hearing loss is primarily caused by the damage or loss of cochlear hair cells, induced by factors such as noise exposure and ototoxic drugs. The deficiency of apoptosis‐inducing gene ***Cidea*** in Cidea KO mice or by delivering ***CRISPR/SlugCas9‐HF*** via AAV to edit ***Cidea*** effectively alleviated hair cell damage and hearing loss caused by neomycin treatment and noise exposure.

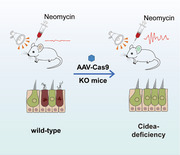

## 1 Introduction

Sensorineural hearing loss (SNHL), the predominant type of hearing loss worldwide, arises primarily from factors such as noise exposure, ototoxic drugs, infections, age‐related degeneration, and hereditary mutations. Aminoglycoside antibiotics, such as neomycin, gentamicin, streptomycin, and kanamycin, constitute a class of broad‐spectrum antibiotics widely employed in clinical practice for the treatment of infections caused by Gram‐negative pathogens. However, their use is limited by ototoxic side effects. Aminoglycoside antibiotics enter cochlear hair cells (HCs) and induce HC loss mainly by increasing reactive oxygen species (ROS) and inducing apoptosis.^[^ ^1^ ^]^ Noise exposure is another common cause of hearing loss, and intense or prolonged exposure to noise can lead to HC loss and thus result in permanent SNHL. Noise exposure induces dysfunction of the stria vascularis, the overproduction of ROS, cochlear inflammation, and the activation of apoptosis, all of which lead to HC death.^[^ ^2^ ^]^

Interestingly, the cell death‐inducing DNA fragmentation factor alpha (DFFA)‐like effector (Cide) family proteins, comprising Cidea, Cideb, and Cidec (alternatively called Fsp27, fat‐specific protein 27), are recognized as apoptosis‐inducing proteins with homology to the CIDE‐N domain of DFFA.^[^ ^3^, ^4^ ^]^ These proteins are closely involved in lipid metabolism, with ***Cide*** gene knockout mice exhibiting a lean phenotype.^[^ ^5^ ^]^ Among them, ***Cidea***, a key member of the CIDE protein family, is highly expressed in brown adipose tissue (BAT), human white adipose tissue, the mammary gland, and the sebaceous gland.^[^ ^6^, ^7^, ^8^, ^9^ ^]^ It plays critical roles in lipid metabolism, storage, and secretion. Additionally, ***Cidea*** can induce DNA fragmentation and apoptosis, which can be inhibited by DFF45.^[^ ^3^ ^]^ The suppression of ***Cidea*** has been shown to protect mouse β‐cells from palmitic acid‐induced apoptosis,^[^ ^10^ ^]^ and in certain tumor‐derived tissues, ***Cidea*** induces apoptosis via the JNK signaling pathway.^[^ ^11^, ^12^ ^]^ Based on these observations, we hypothesize that disruption of ***Cidea*** in the cochlea may protect HCs from apoptosis.

Although several inherited forms of SNHL have been successfully treated with gene therapy,^[^ ^13^, ^14^, ^15^, ^16^, ^17^, ^18^, ^19^, ^20^, ^21^, ^22^, ^23^, ^24^, ^25^, ^26^ ^]^ the treatment of acquired SNHL, caused by ototoxic drugs or noise exposure, remains unsatisfactory due to the lack of an ideal therapeutic target. In this study, we demonstrate that ***Cidea*** plays a crucial role in HC survival and hearing function. Furthermore, we show that targeted disruption of ***Cidea*** using CRISPR‐Cas9 effectively protects HCs from apoptosis induced by neomycin and noise exposure. These findings highlight ***Cidea*** as a promising therapeutic target and provide a potential strategy for the treatment of acquired SNHL.

## 2 Results

### 2.1 Cidea Expression is Specifically Upregulated in Neomycin‐Damaged HCs

**Figure 1:**
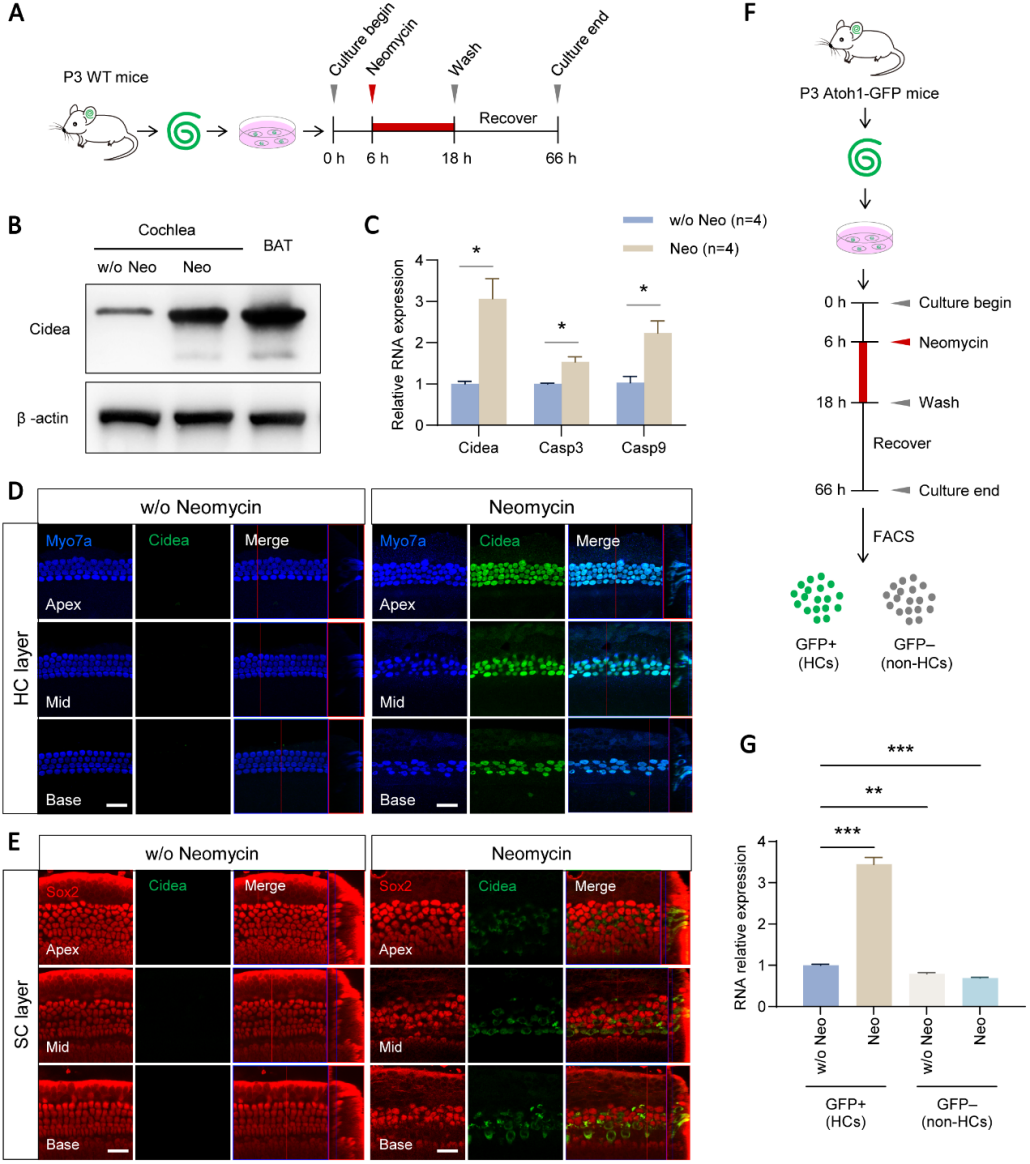
Cidea expression is specifically upregulated in neomycin‐damaged HCs. A) The flow chart of the ex vivo neomycin (Neo) HC damage model. The cochleae were treated with 0.5 mM neomycin. B,C) The protein and mRNA expression levels of Cidea were assessed by Western blot (B) and RT‐qPCR (C). BAT was used as the positive control. w/o, without. D,E) Cidea expression in the cochlea was detected by immunofluorescent staining. Myo7a was used as the HC marker, and Sox2 was used as the SC marker. Scale bar, 20 µm. F,G) *Cidea* expression was assessed by RT‐qPCR after FAC sorting of neomycin‐damaged cochleae from Atoh1‐GFP mice. HCs are GFP+ cells. n = 4 in each group in (G). ^*^ *p* < 0.05, ^**^ *p* < 0.01, ^***^ *p* < 0.001.

To determine whether Cidea is expressed in the cochlea, we extracted total mRNA and protein from cochlear tissue (**Figure** **1**A). Both RT‐qPCR and Western blot analyses revealed minimal basal expression of Cidea (Figure 1B,C). To investigate its regulation under ototoxic stress, we treated isolated cochleae with neomycin ex vivo (Figure 1A). Following treatment, Cidea mRNA and protein levels increased, with mRNA levels increasing approximately threefold (Figure 1B,C). We also examined the expression levels of the other two Cide family members, Cideb and Fsp27, in the cochlea. Cideb was expressed at low levels, while Fsp27 was undetectable (Figure S1A,B, Supporting Information). Notably, Cideb expression remained unchanged after neomycin treatment.

To determine the spatial expression pattern of Cidea in the cochlea, we performed immunofluorescent staining using an anti‐Cidea antibody. Cidea was not detected in untreated cochleae, likely due to its low basal expression. However, following neomycin exposure, robust Cidea expression was observed in HCs (Figure 1D), but not in supporting cells (SCs) (Figure 1E), suggesting a specific role for Cidea in HCs during ototoxic stress.

To confirm these findings, we isolated cochleae from Atoh1‐GFP mice, in which only HCs express GFP (Figure S2A,B, Supporting Information), and treated them with neomycin (Figure 1F). Fluorescence‐activated cell sorting (FACS) analysis revealed that neomycin‐induced Cidea upregulation occurred exclusively in GFP‐positive cells, with no significant increase in GFP‐negative cells (Figure 1G). These results are consistent with our immunofluorescence observations and further support the HC‐specific induction of Cidea following neomycin treatment.

### 2.2 Cidea Promotes Apoptosis in Neomycin‐Damaged HEI‐OC1 Cells

To investigate whether Cidea promotes apoptosis in HCs, we established an in vitro neomycin‐induced damage model using HEI‐OC1 cells (Figure S3A, Supporting Information). Following neomycin treatment, significant upregulation of Cidea expression was observed at both the mRNA and protein levels (Figure S3B,C, Supporting Information). Immunofluorescence staining further showed that Cidea was specifically increased in Myo7a‐positive cells (Figure S3D, Supporting Information), suggesting a potential role of Cidea in HC‐like populations.

To assess the functional role of Cidea, we designed three siRNAs (si620, si683, and si588) to knock down its expression. Three days after transfection of siRNAs into HEI‐OC1 cells, RT‐qPCR analysis revealed that si620 and the combination of si620+si588 achieved the most effective knockdown (Figure S4A, Supporting Information), and si620 was selected for subsequent experiments. Western blot analysis confirmed that si620 significantly reduced Cidea protein levels (Figure S4B, Supporting Information). In parallel, we constructed a plasmid (pCMV‐HA‐Cidea) to overexpress Cidea in HEI‐OC1 cells (Figure S4C,D, Supporting Information). Apoptosis assay using Annexin V and propidium iodide (PI) demonstrated that Cidea overexpression markedly increased apoptotic cell death (Figure S4E,F, Supporting Information), while Cidea knockdown significantly decreased neomycin‐induced apoptosis (Figure S4G,H, Supporting Information). Collectively, these results indicate that Cidea promotes apoptosis in neomycin‐damaged HEI‐OC1 cells, consistent with its known pro‐apoptotic role in other tissues.

### 2.3 Cidea Deficiency Protects HCs from Neomycin‐Induced Damage Ex Vivo

**Figure 2:**
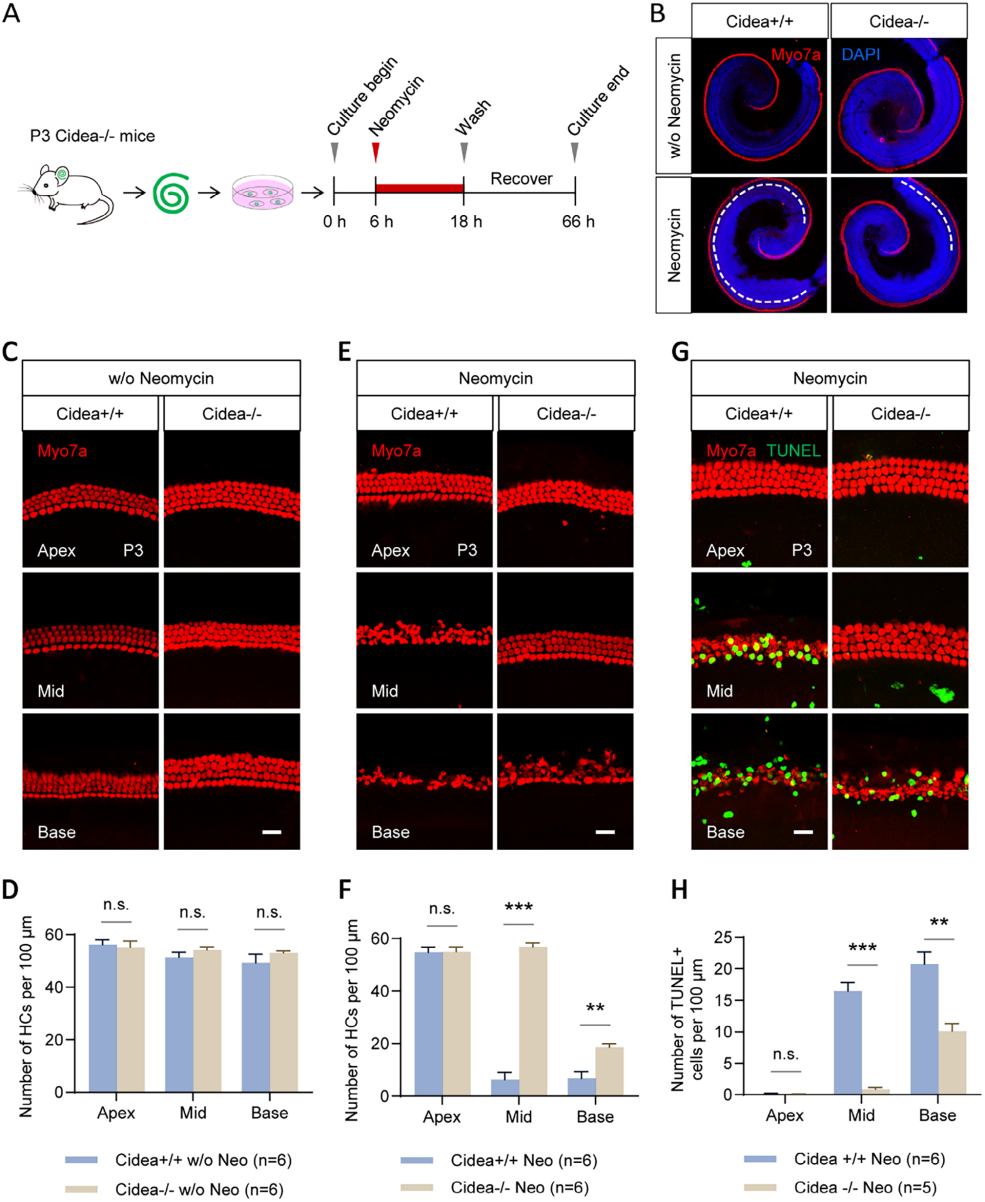
Cidea deficiency protects HCs from neomycin‐induced damage ex vivo. A) The flow chart of the *ex vivo* neomycin HC damage model. The cochleae were treated with 0.5 mM neomycin. B–F) Cochleae of WT (*Cidea*+/+) mice and *Cidea*‐/‐ mice were dissected and immunostained (B,C,E), and the HC number in each turn was quantified (D,F). *Cidea*+/+ mice were used as controls. Myo7a was used as the HC marker. The dashed lines in (B) show the cochlear regions with HC damage. Scale bar, 20 µm in (C, E). G,H) TUNEL+ HCs were stained (G) and quantified (H) in *Cidea*‐/‐ mouse cochleae and compared with *Cidea*+/+ mice after neomycin treatment ex vivo. Scale bar, 20 µm in (G). n.s., not significant. ^**^ *p* < 0.01, ^***^ *p* < 0.001.

To further investigate the role of Cidea, we generated Cidea knockout (Cidea‐/‐) mice. Cochleae were isolated and treated with neomycin *ex vivo* (**Figure** **2**A). Under normal conditions, Cidea deficiency did not affect HC morphology or number in P3 mouse cochleae (Figure 2B–D; Table S2, Supporting Information). Following neomycin treatment, Cidea deficiency significantly preserved HCs in both the middle turn (56.90 ± 1.43 HCs per 100 µm) and basal turn (18.62 ± 1.36 HCs per 100 µm) compared with WT mice (6.30 ± 2.82 HCs per 100 µm in the middle turn and 6.85 ± 2.56 HCs per 100 µm in the basal turn) (Figure 2B,E,F; Table S2, Supporting Information). Notably, HCs in the middle turn were nearly completely protected.

Consistent with the HC survival data, TUNEL assay revealed that Cidea deficiency significantly reduced apoptosis in the middle turn (0.84 ± 0.27 TUNEL+ cells per 100 µm) and basal turn (10.13 ± 1.14 TUNEL+ cells per 100 µm), compared to WT cochleae (16.46 ± 1.39 TUNEL+ cells per 100 µm in middle turn and 20.78 ± 1.89 TUNEL+ cells per 100 µm in basal turn) (Figure 2G,H; Table S3, Supporting Information). These results indicate that Cidea promotes HC apoptosis ex vivo and that its loss confers protection against neomycin‐induced ototoxicity.

### 2.4 Cidea Deficiency Protects HCs and Hearing Function from Neomycin‐Induced Damage In Vivo

**Figure 3:**
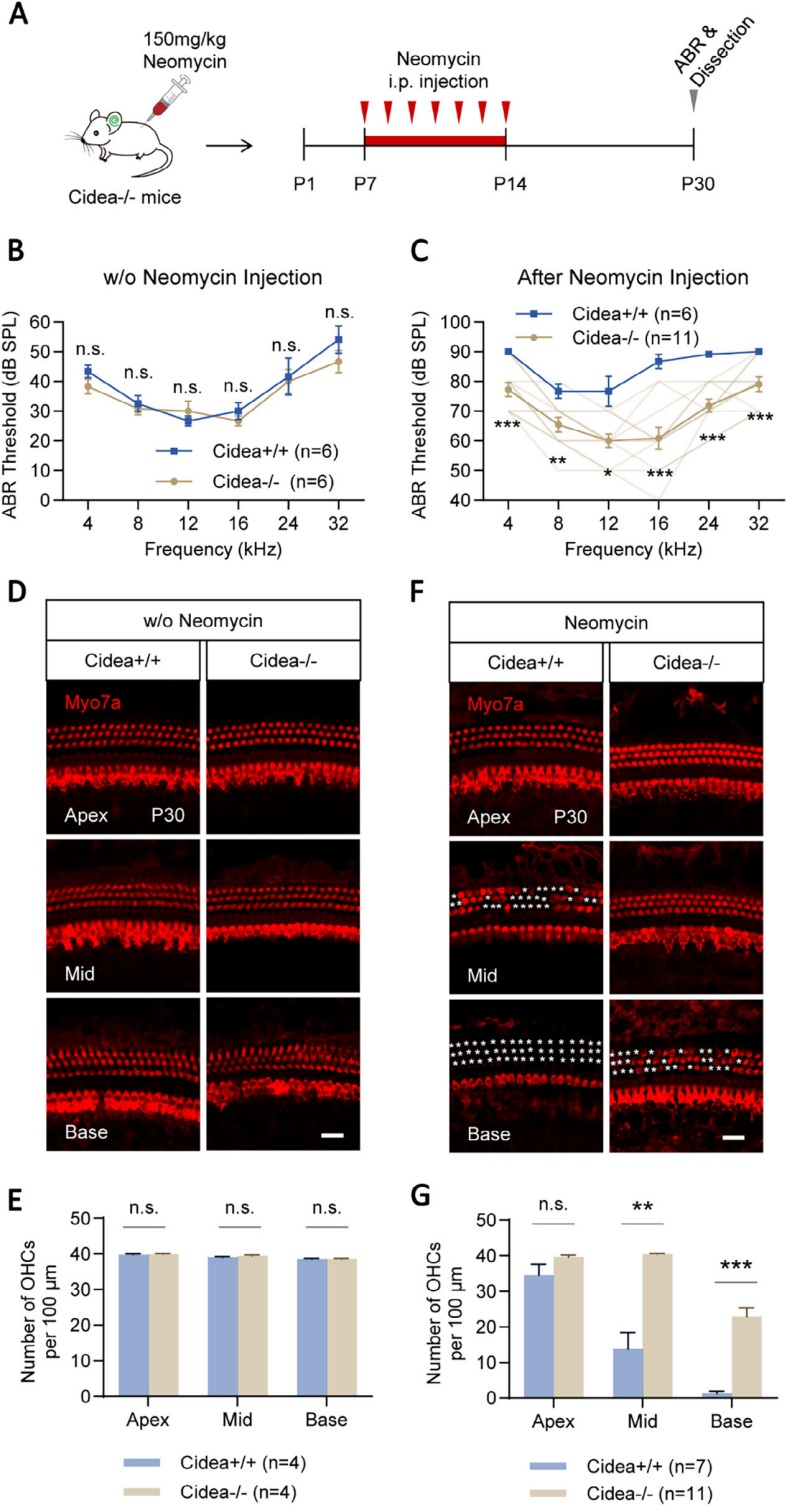
Cidea deficiency protects HCs and hearing function from neomycin‐induced damage in vivo. A) The flow chart of the in vivo neomycin HC damage model. Mice were i.p. injected with 150 mg neomycin per kg body weight. B,C) ABR hearing thresholds in P30 *Cidea*‐/‐ mice were measured without (w/o) neomycin (B) and after neomycin i.p. injection in vivo (C). WT *Cidea*+/+ mice were used as controls. The lighter yellow lines in (C) represent the ABR threshold of individual Cidea‐/‐ mice. D–G) Cochlear HCs were immunostained (D,F) and quantified (E,G) without (w/o) neomycin (D,E) or after neomycin i.p. injection in vivo (F,G). The white asterisks in (F) indicate the lost HCs. Scale bar, 20 µm in (D) and (F). n.s., not significant. ^*^ *p* < 0.05, ^**^ *p* < 0.01, ^***^ *p* < 0.001.

Next, we established an in vivo neomycin‐induced damage model by i.p. injection of neomycin (150 mg kg^−1^) into Cidea‐/‐ mice from P7 to P14 for seven consecutive days, and assessed auditory function at P30 (**Figure** **3**A). Cidea deficiency alone did not affect hearing in untreated P30 mice, as indicated by comparable ABR thresholds between Cidea‐/‐ and WT controls (Figure 3B; Table S4, Supporting Information). However, following neomycin exposure, Cidea‐/‐ mice exhibited significantly preserved hearing, with ABR threshold shifts reduced by 11–26 dB on average compared to WT mice (Figure 3C; Table S4, Supporting Information).

Morphological analysis showed that Cidea deficiency did not alter the structure or number of cochlear HCs in untreated P30 mice (Figure 3D,E; Table S5, Supporting Information). In contrast, after neomycin treatment, Cidea‐/‐ mice were significantly protected from outer HC (OHC) loss, particularly in the middle turn (40.45 ± 0.21 OHCs per 100 µm) and basal turn (22.93 ± 2.45 OHCs per 100 µm) of the cochlea, compared to WT mice (13.82 ± 4.60 OHCs per 100 µm in the middle turn and 1.21 ± 0.74 OHCs per 100 µm in the basal turn) (Figure 3F,G; Table S5, Supporting Information). Notably, this protective effect was not observed in Cidea+/‐ heterozygous mice (Figure S5A–C and Table S5, Supporting Information). These findings demonstrate that complete Cidea deficiency confers significant protection against neomycin‐induced HC damage and hearing loss in both ex vivo and in vivo models.

### 2.5 Cidea Deficiency Protects HCs and Hearing Function From Noise‐Induced Damage

**Figure 4:**
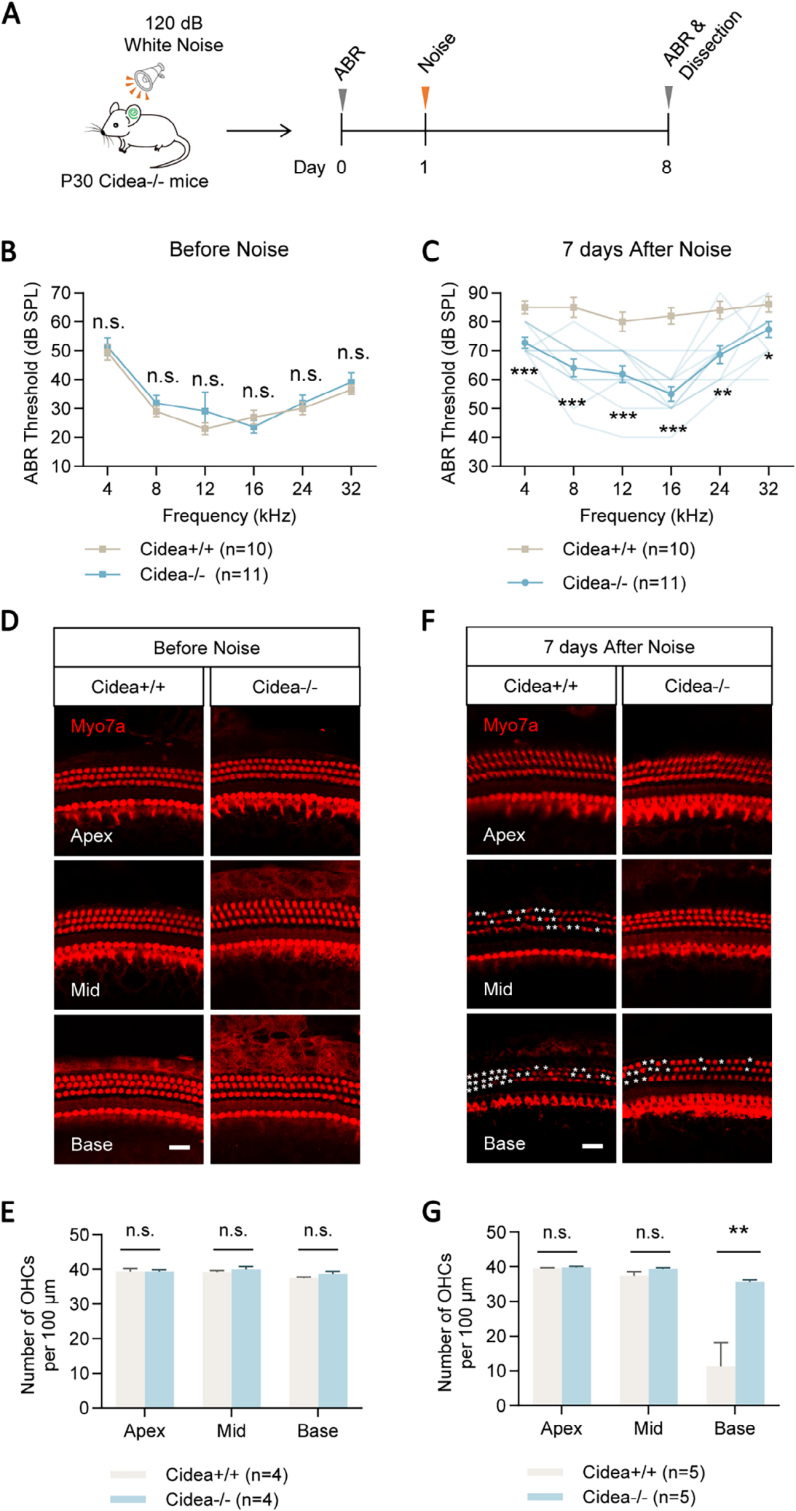
Cidea deficiency protects HCs and hearing function from noise‐induced damage. A) The flow chart of the noise‐induced HC damage model. Mice were exposed to 120 dB white noise for 2 h. B,C) ABR hearing thresholds in P30 *Cidea*‐/‐ mice were measured before (B) and 7 days after (C) noise exposure. WT *Cidea*+/+ mice were used as controls. The lighter green lines in (C) represent the ABR threshold of individual Cidea‐/‐ mice. D–G) Cochlear HCs were immunostained (D,F) and quantified (E,G) before (D,E) and 7 days after (F,G) noise exposure. The white asterisks in (F) indicate the lost HCs. Scale bar, 20 µm in (D) and (F). n.s., not significant. ^*^ *p* < 0.05, ^**^ *p* < 0.01, ^***^ *p* < 0.001.

Given that noise exposure is another major cause of SNHL, we hypothesized that Cidea deficiency may also confer protection against acoustic trauma. To test this, Cidea‐/‐ mice were exposed to 120 dB white noise for 2 h, and ABR thresholds were measured at 7 days post‐exposure (**Figure** **4**A). Prior to noise exposure, ABR thresholds were comparable between Cidea‐/‐ and WT mice (Figure 4B). However, at 7 days after noise exposure, Cidea‐/‐ mice showed significantly lower ABR threshold shifts (9–27 dB reduction) compared to WT mice (Figure 4C; Table S6, Supporting Information).

To assess cochlear morphology, we performed immunofluorescent staining on cochlear whole mounts. Before noise exposure, Cidea deficiency did not alter the structure or number of cochlear HCs in P30 mice (Figure 4D,E; Table S7, Supporting Information), as identified in the P30 mice cochlea without neomycin injection (Figure 3D,E; Table S5, Supporting Information). Seven days after noise exposure, quantification showed significantly reduced OHC loss in the basal turn of Cidea‐/‐ mice (35.69 ± 0.51 OHCs per 100 µm) compared to WT controls (11.28 ± 6.82 OHCs per 100 µm) (Figure 4E,F; Table S7, Supporting Information). These results closely mirror the protective phenotype observed in the neomycin‐induced damage model. Taken together, these findings suggest that Cidea deficiency protects against both noise‐ and drug‐induced hearing loss and HC degeneration in vivo.

### 2.6 In Vivo Disruption of Cidea Using CRISPR‐Cas9 Suggests Therapeutic Potential for SNHL

The results described above suggest that Cidea may serve as a promising therapeutic target for the treatment of acquired SNHL. To evaluate this possibility, we employed SlugCas9‐HF, a high‐fidelity variant of Cas9, to disrupt the Cidea gene. Three sgRNAs were designed to target exon 2 of Cidea (Figure S6A, Supporting Information). Following transfection with Cas9 and sgRNA‐expressing plasmids (Figure S6B, Supporting Information), genomic DNA was extracted and analyzed by targeted deep sequencing. Among the three candidates, sgRNA2 showed the highest editing efficiency (Figure S6C,D, Supporting Information) and was selected for subsequent experiments.

We next assessed genome editing efficiency in vivo. An AAV vector of serotype PHP.eB was chosen as the delivery vector due to its high transduction efficiency and specificity for cochlear HCs. To validate its targeting properties, we injected 1.5 × 10^10^ vg of PHP.eB‐EGFP AAV into the cochleae of P1 mice via the RWM. At P7, strong EGFP expression was observed specifically in HCs, but not in SCs, as confirmed by immunofluorescence staining (Figure S6E, Supporting Information), demonstrating the vector's high specificity and efficiency.

We then used AAV‐PHP.eB to deliver Cas9 and sgRNA2. Approximately 2.9 × 10^10^ vg PHP.eB‐SlugCas9‐HF‐sgRNA2 virus was injected into the cochleae of P1 WT mice via the RWM. Western blot showed a significant decrease in Cidea protein expression levels (Figure S5F, Supporting Information), indicating effective gene disruption and supporting the feasibility

### 2.7 Cidea Genome Editing Protects HCs and Alleviates Hearing Loss in Neomycin‐Treated Mice

**Figure 5:**
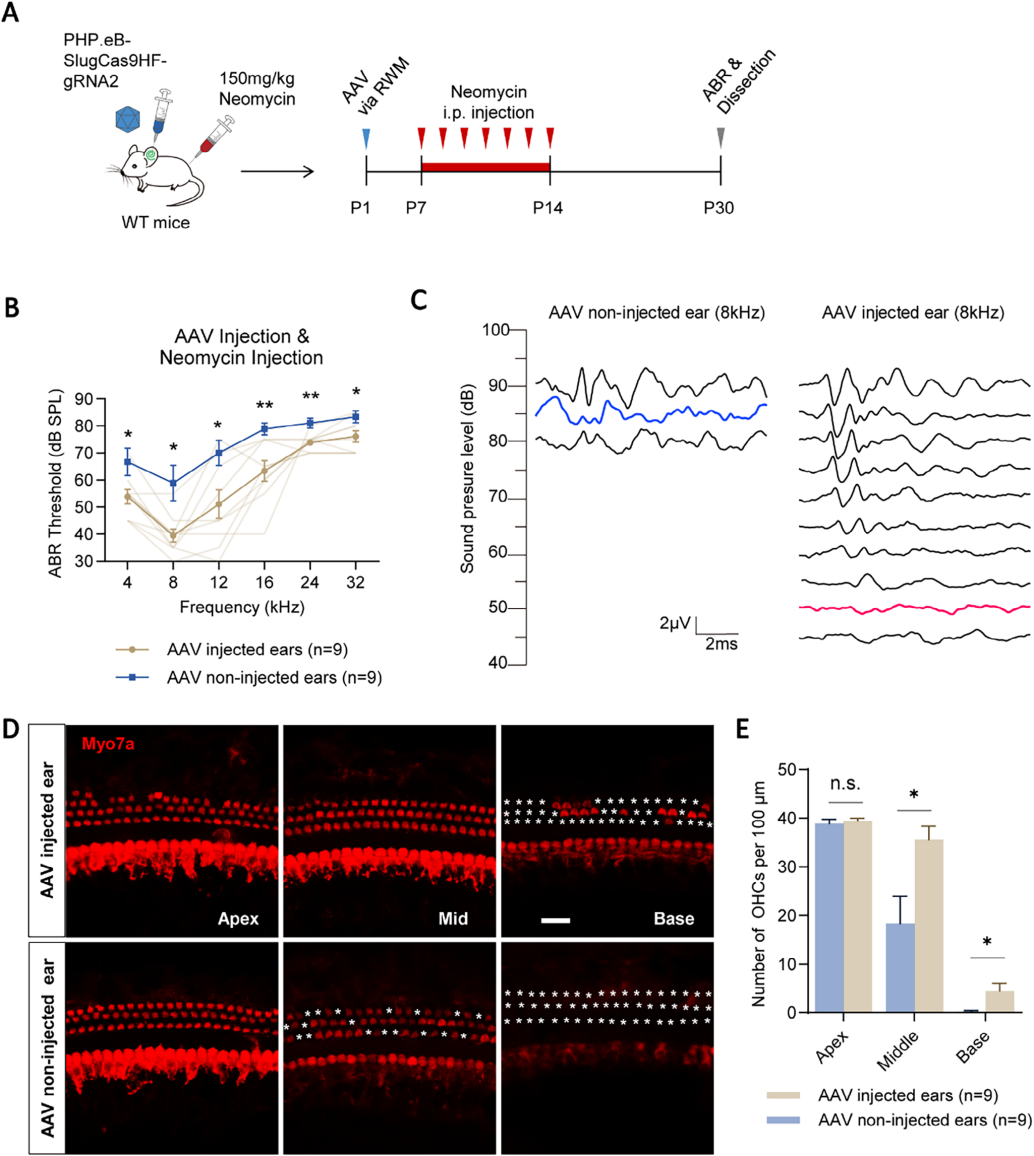
Cidea genome editing protects HCs and alleviates hearing loss in neomycin‐treated mice. A) The flow chart of the experimental design. A total of 1.5 µL PHP.eB‐SlugCas9‐HF‐sgRNA2 virus with a titer of 1.93 × 10^13^ vg mL^−1^ was injected into the left ear of P1 WT mice through the RWM for editing *the Cidea* gene. Neomycin (150 mg kg^−1^ body weight) was then i.p. injected daily from P7 to P14 to induce HC damage. B) Comparison of the ABR thresholds of the AAV injected and non‐injected ears at P30. The lighter yellow lines in (B) represent the ABR threshold of individual AAV injected ears. C) Representative individual ABR results from the most protected AAV‐injected ear and non‐injected ear at 8 kHz after neomycin damage at P30. The blue and red traces indicate the thresholds. D,E) The cochleae of the AAV injected and non‐injected ears were dissected and immunostained (D), and the HC number was quantified (E). Myo7a was used as the HC marker. The white asterisks in (D) indicate the lost HCs. Scale bar, 20 µm. n.s., not significant. ^*^ *p* < 0.05, ^**^ *p* < 0.01.

To evaluate the therapeutic potential of Cidea genome editing for hearing protection, we injected the PHP.eB‐SlugCas9‐HF‐sgRNA2 virus (≈2.9 × 10^10^ vg) into the left cochlea of P1 WT mice via the RWM. The right ear served as an untreated internal control. Mice were then i.p. administered neomycin (150 mg kg^−1^ body weight) daily from P7 to P14. ABR thresholds were assessed, and cochleae were collected for immunostaining at P30 (**Figure** **5**A).

ABR analysis revealed that Cidea editing significantly attenuated the hearing loss caused by neomycin. Threshold elevations were reduced across all tested frequencies, with average improvements of 7–19 dB in the AAV‐injected ears compared to the contralateral non‐injected controls (Figure 5B; Table S8, Supporting Information). A representative 8 kHz ABR waveform from the most protected ear showed a 35 dB threshold reduction (Figure 5C).

To assess HC survival, we performed immunofluorescence staining on dissected cochleae. Quantification revealed significantly reduced OHC loss in the AAV‐injected ears—35.56 ± 2.81 OHCs per 100 µm in the middle turn and 4.44 ± 1.61 OHCs per 100 µm in the basal turn—compared to the non‐injected ears, which exhibited 18.33 ± 5.62 and 0.22 ± 0.22 OHCs per 100 µm, respectively (Figure 5D,E; Table S9, Supporting Information). Notably, HCs in the middle turn were almost completely preserved. Together, these results demonstrate that Cidea disruption via PHP.eB‐SlugCas9‐HF‐sgRNA2 virus promotes HC survival and protects hearing function in an in vivo model of neomycin‐induced ototoxicity, indicating the feasibility of this strategy for Cidea‐targeted gene therapy in the cochlea.

### 2.8 Cidea Genome Editing Protects HCs and Alleviates Hearing Loss in Noise‐Treated Mice

**Figure 6:**
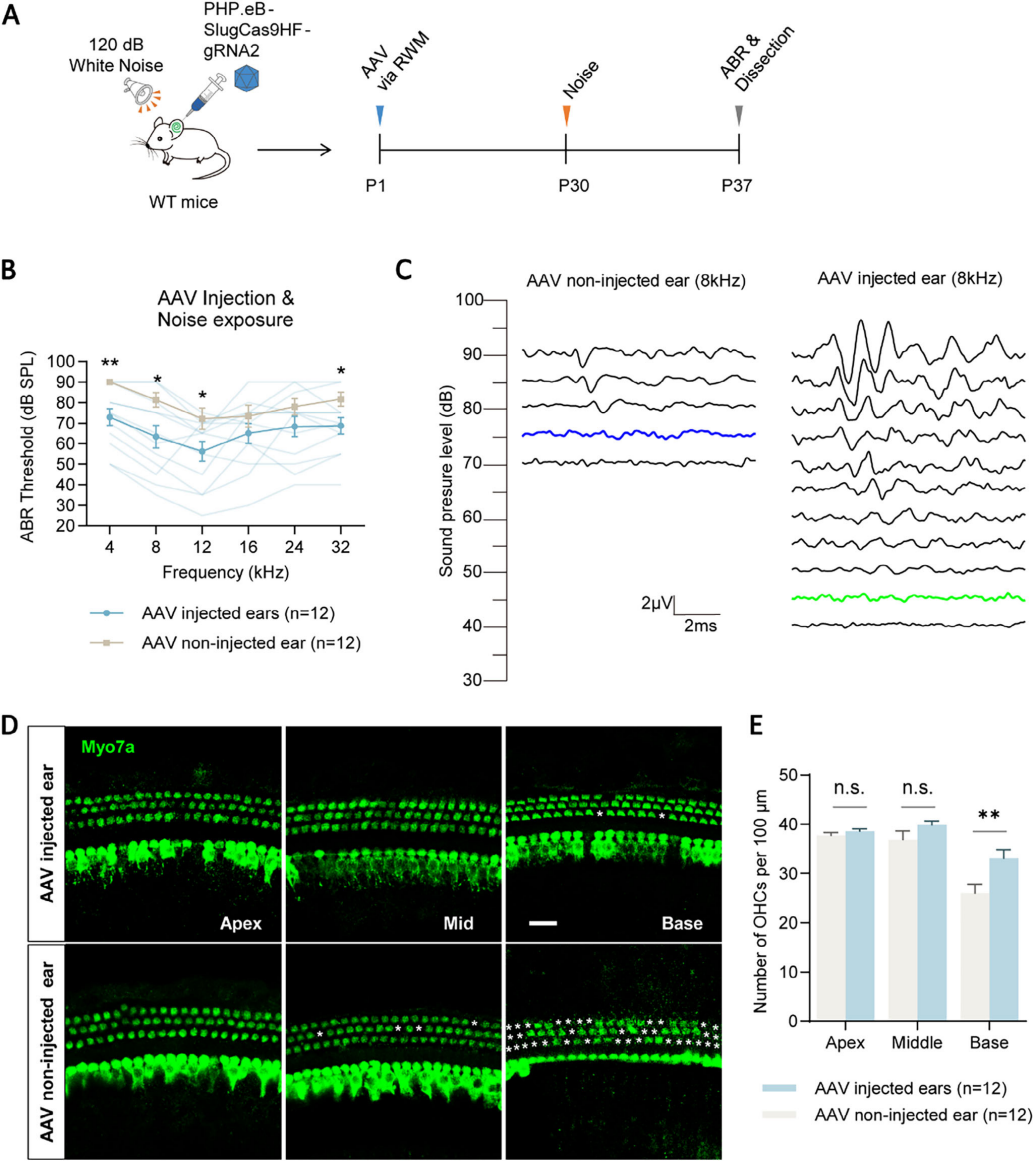
Cidea genome editing protects HCs and alleviates hearing loss in noise‐treated mice A) The flow chart of the experiment. A total of 1.5 µL PHP.eB‐SlugCas9HF‐sgRNA2 virus with a titer of 1.93 × 10^13^ vg mL^−1^ was injected into the left ear of P1 WT mice through the RWM for editing the *Cidea* gene. Mice were then exposed to 120 dB white noise for 2 h. B) Comparison of the ABR thresholds of AAV injected and non‐injected ears 7 days after noise exposure. The lighter green lines in (B) represent the ABR threshold of individual AAV injected ears. C) Representative individual ABR results from the most protected AAV injected ear and non‐injected ear at 8 kHz after noise exposure at P30. The blue and green traces indicate the thresholds. D,E) The cochleae of the AAV injected and non‐injected ears were dissected and immunostained (D), and the HC number was quantified (E). Myo7a was used as the HC marker. The white asterisks in (D) indicate the lost HCs. Scale bar, 20 µm. n.s., not significant. ^*^ *p* < 0.05, ^**^ *p* < 0.01.

We next evaluated the efficacy of Cidea gene editing in preventing hearing loss caused by acoustic trauma. PHP.eB‐SlugCas9‐HF‐sgRNA2 virus (≈2.9 × 10^10^ vg) was injected into the left cochlea of P1 WT mice via the RWM to achieve in vivo editing of Cidea in HCs. At P30, the mice were exposed to 120 dB white noise for 2 h, followed by ABR testing at 7 days post‐exposure (**Figure** **6**A).

Consistent with the neomycin damage model, Cidea editing significantly mitigated the elevation of ABR thresholds following noise exposure. Significant threshold improvements were observed at 4, 8, 12, and 32 kHz, with an average reduction of 13–18 dB in the AAV‐injected ears compared to the non‐injected controls (Figure 6B; Table S10, Supporting Information). A representative 8 kHz ABR waveform from the most protected ear showed a 30 dB threshold reduction (Figure 6C).

We then dissected the cochleae and performed immunofluorescent staining to quantify HC survival. In the basal turn, the AAV‐injected ears showed significantly greater preservation of OHCs (33.08 ± 1.73 OHCs per 100 µm) compared to the non‐injected ears (26.00 ± 1.84 OHCs per 100 µm) (Figure 6D,E; Table S11, Supporting Information). These findings demonstrate that Cidea editing via PHP.eB‐SlugCas9‐HF‐sgRNA2 promotes HC survival and preserves auditory function in an in vivo model of hearing loss caused by noise exposure.

## 3 Discussion

SNHL is caused by many factors, including noise exposure, ototoxic drugs, infection, aging, and genetics, and although SNHL is a common clinical complaint, there is still a lack of effective therapeutic options.^[^ ^28^ ^]^ Loss of or damage to cochlear HCs is the primary cause of SNHL, and thus protecting HCs is the primary focus of research into SNHL treatments. The accumulation of ROS and the induction of apoptosis play important roles in this process,^[^ ^29^, ^30^ ^]^ and thus antioxidants and the inhibition of apoptosis have been studied for attenuating SNHL.^[^ ^31^ ^]^ Extensive efforts were made to preserve hearing through interventions targeting key molecular pathways using small molecules, gene therapy, or RNA interference (RNAi)^[^ ^32^, ^33^, ^34^ ^]^ and through editing of BDNF, c‐myb, Neurotrophin‐3 (NT‐3), and apoptosis‐associated genes such as *XIAP*, *Htra2*, *Bcl‐xl*, and *SIRT1* using AAV and CRISPR/Cas9 system.^[^ ^35^, ^36^, ^37^, ^38^, ^39^, ^40^, ^41^ ^]^ However, the efficacy of these therapeutic strategies is limited, only partially mitigating HC damage and hearing loss, which may be because SNHL is caused by multiple factors and the mechanism involves multiple pathways and genes. Therefore, the specific mechanisms and more therapeutic targets of SNHL need to be studied.

Cide family proteins—Cidea, Cideb, and Fsp27—were initially discovered to be highly homologous to the N‐terminus of DFF45 and were shown to be apoptosis‐inducing factors.^[^ ^3^ ^]^ However, whether they are expressed and play roles in the cochlea has not been studied yet. Here, we found that Cidea and Cideb were both expressed at low levels in the cochlea, while Fsp27 showed no expression in the cochlea. Among them, only the expression of Cidea was significantly increased after cochlear injury, which suggests that Cidea might provide a cochlear injury signal, and inhibition of Cidea thus might be useful as a new therapeutic strategy for HC protection and hearing loss. The apoptosis‐inducing role of Cidea was also confirmed in this study, as previously reported.^[^ ^10^, ^11^, ^12^ ^]^ Cideb, with a low cochlear expression level, may also play roles in the cochlea that are unrelated to cochlear injury, and this needs further study in the future.

In this study, we found that hearing function and HC morphology were not influenced by Cidea deficiency, and that Cidea deficiency significantly alleviated hearing loss and HC damage caused by neomycin treatment and noise exposure, which suggests that Cidea might be a useful therapeutic target. We also found that the hearing and cochlear phenotypes of *Cidea*+/‐ mice were similar to WT mice, which means that heterozygous loss of *Cidea* is not sufficient for its protective roles. It must be noted that even homozygous loss of *Cidea* could not fully protect against hearing loss and HC damage in either the neomycin or noise damage models, which suggests that there are other pathways unrelated to Cidea that also play a role in this process.

Here, we used SlugCas9‐HF, which is reported to be an effective and high‐fidelity gene therapy tool^[^ ^42^, ^43^, ^44^ ^]^ for editing the *Cidea* gene. We designed three sgRNAs for *Cidea* and chose the most efficient sgRNA2 for the subsequent experiments, and PHP.eB AAV was used as the delivery tool for efficient and specific HC infection. Editing of *Cidea* in HCs by injecting PHP.eB‐SluCa9‐HF‐sgRNA2 through the RWM achieved the goal of hearing and HC protection to some extent. The injected ear showed a 7–19 dB improvement in ABR thresholds compared with the non‐injected ear in both the neomycin and noise damage models. HCs in the middle turn were almost fully protected in the injected ear after neomycin damage, while HCs in the basal turn were only partially protected in the injected ear in both the neomycin and noise damage models. However, the ABR thresholds did not recover to normal, and HCs were not fully protected from damage. On one hand, this may be because *Cidea* was only knocked down, not knocked out, by gene editing in the cochlea in vivo, and a more efficient Cas9 and sgRNA and an AAV with higher infection efficiency may improve the therapeutic effect in the future. On the other hand, even in *Cidea* knockout mice, the ABR thresholds did not recover to normal, and HCs were not fully protected from damage; perhaps simultaneous inhibition of Cidea and other HC damage pathways can achieve complete HC protection.

It is noteworthy that the protective effect of Cidea deficiency appears to slightly differ between the neomycin‐induced and noise‐induced hearing damage models, both in KO mice and in gene editing cases. We propose that this discrepancy may be partly attributable to the fact that noise exposure not only causes HC loss, but also leads to auditory nerve degeneration and synaptic impairment^[^ ^29^ ^]^—effects that Cidea deficiency in HCs cannot mitigate. Additionally, the two injury models result in differing patterns of HC loss, particularly in the middle turn of the cochlea. Given the absence of significant middle‐turn HC loss in the noise damage model, the protective influence of Cidea deficiency did not achieve statistical significance.

Cidea, a member of the cell death‐inducing Cide family proteins, was initially reported to activate apoptosis by inducing DNA fragmentation in a manner that was inhibited by DFF45, but not by caspase inhibitors.^[^ ^3^ ^]^ The expression of Cidea was found to be upregulated when apoptosis occurs under diverse physiological and pathological contexts, including skeletal muscle post‐burn injury,^[^ ^45^ ^]^ kidneys following ischemia‐reperfusion or cisplatin‐induced damage,^[^ ^46^ ^]^ and pancreatic β‐cells exposed to free fatty acids (FFA).^[^ ^10^ ^]^ Moreover, while Cidea is downregulated in several tumor types, its expression is upregulated in response to apoptosis‐inducing anti‐tumor treatments.^[^ ^11^, ^12^, ^47^, ^48^ ^]^ Several studies have established Cidea as a downstream target of PPARγ^[^ ^11^ ^]^ and FoxO1^[^ ^10^ ^]^ in pro‐apoptotic responses. Furthermore, ectopic expression of Cidea has been shown to trigger a series of downstream events, including JNK activation, cell cycle arrest, increased p53 acetylation, decreased STAT3 phosphorylation, and reduced IL‐6 release.^[^ ^11^, ^12^ ^]^ However, the precise signaling pathway involving Cidea remains to be elucidated. Our findings regarding Cidea upregulation in damaged cochlear HCs corroborate the existing literature mentioned above. Nevertheless, the precise mechanism through which Cidea deficiency protects HC from damage by neomycin and noise warrants additional study.

In the future, we believe that Cidea can be a useful therapeutic target for treating SNHL in the clinic. To achieve this, more efficient *Cidea* editing approaches and combinations of gene therapy strategies should be developed, and the long‐term safety of the therapeutic system should be evaluated.

## 4 Experimental Section

### Animals and the In Vivo HC Damage Model

Cidea‐deficient mice (*Cidea*‐/‐ mice) were a gift from Professor Peng Li of Tsinghua University,^[^ ^7^ ^]^ and Atoh1‐GFP mice^[^ ^27^ ^]^ of both sexes were used in this study. All animal experiments were conducted in strict accordance with protocols approved by the Animal Care and Use Committee of Southeast University (experiment number: SEU‐IACUC‐20250902004) following the Guide for the Care and Use of Laboratory Animals outlined by the National Institutes of Health. Every effort was made to minimize animal usage and to alleviate potential distress in the experimental procedures.

Genotyping of Cidea‐deficient mice was performed as described previously.^[^ ^7^, ^9^ ^]^ Briefly, mouse tail tips were digested in 500 µL lysis buffer (0.4 m NaCl, 0.1 m Tris‐HCl, pH 8.5, 5 mM EDTA, 0.5% SDS, 0.2 mg mL^−1^ proteinase K, 0.1 mg mL^−1^ RNase A) at 55 °C overnight. The lysate was mixed with 500 µL phenol: chloroform, vigorously vortexed, and centrifuged at 12 000 × g for 15 min at 4 °C. The supernatant was combined with 600 µL of ice‐cold isopropanol to precipitate the genomic DNA, followed by centrifugation at 12 000 × g for 15 min at 4 °C. The genomic DNA pellet was washed with 70% ethanol, centrifuged at 7000 × g for 10 min at 4 °C, air‐dried, and resuspended in 50 µL of nuclease‐free water. The genotyping PCR amplification was carried out using a 20 µL reaction mixture containing 0.5 µL genomic DNA template, 2 µL primers, 10 µL PCR mix (Vazyme, p131‐02), and nuclease‐free water to volume. The thermal cycling conditions were: initial denaturation at 94 °C for 3 min; 30 cycles of denaturation at 94 °C for 30 s, annealing at 60 °C for 30 s, and extension at 72 °C for 3 min. The primer sequences were: forward: 5′‐GCC CCA GGC CTG GAC TCT GAG CTA G‐3′; reverse: 5′‐GGC ACA GAA CCA AAA CCC CGA AGT G‐3′.

To establish the in vivo neomycin HC damage model, postnatal day 7 (P7) mice were given an intraperitoneal (i.p.) injection of neomycin (Sigma, #N6386, 150 mg kg^−1^ body weight) daily from P7 to P14 for seven consecutive days. At P30, the mice were subjected to ABR assessment and then sacrificed for cochlear dissection.

To induce in vivo HC damage by noise exposure, P30 mice were placed in a soundproof chamber and subjected to a 2 h exposure to 120 dB white noise. ABR thresholds were assessed before and 7 days after noise exposure.

### Cochlear Explant Culture and the Ex Vivo HC Damage Model

The cochlear basilar membranes of wild‐type (WT) mice, *Cidea*‐/‐ mice, and Atoh1‐GFP mice were dissected in cold HBSS, adhered to round glass slides (Biosharp, #BS‐09‐RC) that had been pre‐coated with Cell‐Tak (Corning, #354240), and cultured in advanced DMEM/F‐12 medium (Gibco, #12634010) containing 1% N‐2 (Thermo Fisher, #175020‐48), 2% B‐27 (Thermo Fisher, #17504044), 10 ng mL^−1^ b‐FGF (Sigma, #F0291), 20 ng mL^−1^ EGF (Sigma, #E9644), 50 ng mL^−1^ IGF (Sigma, #I8779), and 1% ampicillin (Beyotime, #ST008) with 5% CO_2_ at 37 °C. At 6 h post‐culture initiation, 0.5 mM neomycin (Sigma, #N6386) was added for 12 h, and after a 48 h recovery period, the cochleae were harvested for subsequent experiments.

### Flow Cytometry

Cochleae from Atoh1‐GFP mice were dissociated with 0.125% trypsin/EDTA (Gibco, #15050065) at 37 °C for 8 min and separated into single cells by pipetting up and down several times, followed by percolating through a 4‐cell strainer with a 40 µm pore size (BD Biosciences, #352340). Dissociated cells were sorted into GFP+ and GFP– cells on a BD FACS Aria III (BD Biosciences), utilizing the GFP channel for discrimination.

### Immunofluorescence Analysis and Image Acquisition

Cochleae were dissected from neonatal mice using sharp forceps in ice‐cold HBSS, followed by fixation in 4% paraformaldehyde (PFA) at room temperature for 1 h. The cochleae of P30 mice were fixed in 4% PFA for 1 h, followed by a 3‐day decalcification treatment using 0.5 M EDTA at room temperature before dissection in HBSS. Following fixation and dissection, all samples were blocked at room temperature for 1 h in a blocking solution composed of 5% donkey serum, 1% bovine serum albumin (BSA), 0.5% Triton X‐100, and 0.02% sodium azide, prepared in PBS at pH 7.4. Samples were then applied to an overnight incubation with primary antibodies suspended in PBT1 (2.5% donkey serum, 1% BSA, 0.1% Triton X‐100, and 0.02% sodium azide, prepared in PBS at pH 7.4) at 4 °C. Primary antibodies included anti‐Myo7a (Proteus Bioscience, #25‐6790), anti‐Sox2 (R&D Systems, #AF2018), and anti‐Cidea (provided by Prof. Peng Li, Tsinghua University; Proteintech, #13170‐1‐AP). After three washes with PBST buffer (PBS containing 0.1% Triton X‐100), the samples were incubated for 1 h at room temperature with either fluorescent‐conjugated secondary antibodies (Invitrogen) or phalloidin (Invitrogen, #A22281) suspended in PBT2 (1% BSA and 0.1% Triton X‐100 prepared in PBS at pH 7.4). Following additional washes with PBST, the cochleae were coverslipped with a commercial antifade mounting agent (DAKO, #S3023). Apoptotic cells were detected using a TUNEL Kit (Invitrogen, #C10617) following the manufacturer's protocol. High‐resolution images were captured and analyzed with a confocal microscope (Zeiss LSM 710) and ImageJ software, respectively.

### Western Blot

Mouse cochlear tissues and BAT tissues were homogenized in a mixture of RIPA lysis buffer (200 µL, Epizyme, #PC101) and 50× protease inhibitor cocktail (4 µL, Roche, #04693132001). For HEI‐OC1 cells, the supernatant was aspirated, and cells were scraped off in 200 µL ice‐cold RIPA buffer. Lysates were mixed with 5× SDS loading buffer (Epizyme, #LT101), denatured at 98 °C for 10 min, and resolved on a 10% SDS‐PAGE gel (Epizyme, #PG112). Proteins were subsequently transferred to a PVDF membrane with a 0.45 µm pore size (Millipore, #IPVH00010). Following a 2 h blockage at room temperature with 5% skim milk in TBST (Tris‐buffered saline containing 0.1% Tween‐20), the membrane was probed with primary antibodies against β‐actin (Abmart, #P30002M), GAPDH (Abmart, #M20006), Cidea (provided by Prof. Peng Li, Tsinghua University; Proteintech, #13170‐1‐AP), and HA tag (Abmart, #M20003) at 4 °C overnight. After three washes in TBST for 5 min each, the PVDF membranes were exposed to HRP‐conjugated secondary antibodies (goat anti‐rabbit/mouse, Abmart, #M21001/M21002) for a 2 h incubation at room temperature. Visualization of the protein bands was achieved by employing a SuperPico ECL Chemiluminescence Kit (Vazyme, #E422) for detection, with image acquisition performed on a Tanon 2500R system.

### RNA Extraction and Reverse Transcription Quantitative PCR (RT‐qPCR)

Total RNA was isolated from mouse cochleae using TRIzol reagent (Invitrogen, #15596026) and was reverse transcribed into cDNA with a RevertAid First Strand cDNA Synthesis Kit (Thermo Scientific, #K1621). Quantification of gene expression levels was subsequently conducted with the FastStart Universal SYBR Green Master (ROX) mix (Roche, #4913914001) on a CFX96 Real‐Time PCR System (Bio‐Rad). The relative gene expression was calculated with the comparative 2^−ΔΔCT^ method for normalization using the endogenous reference gene *Gapdh*. Primer sequences for all analyzed genes are provided in Table S1 (Supporting Information).

### Auditory Brainstem Response (ABR) Measurements

P30 mice were anesthetized via i.p. administration of sodium pentobarbital (100 mg kg^−1^ body weight, 0.01 g mL^−1^ concentration). Subdermal needle electrodes were positioned at the cranial vertex, the postauricular region ipsilateral to the stimulated ear, and the thigh as a ground reference. ABR thresholds were assessed in a soundproof chamber using a Tucker–Davis Technologies RZ6 workstation controlled by SigGen32 software. Tone bursts at frequencies of 4, 8, 12, 16, 24, and 32 kHz were presented in an open‐ or closed‐field configuration. Threshold determination was based on the minimal sound intensity required to elicit ABR waveform responses.

### AAV Vector Production and Cochlear Delivery

Three single‐guide RNAs (sgRNAs1‐3) targeting distinct regions of the *Cidea* gene locus were designed for genome editing, and the SlugCas9‐HF nuclease was included in the plasmid with one of the sgRNAs (Figure S6A,B, Supporting Information). The most efficient SlugCas9‐HF‐sgRNA2 construct was cloned into an AAV vector of serotype PHP.eB (PHP.eB‐SlugCas9‐HF‐sgRNA2). Viral packaging was performed by Taitool Bioscience (Shanghai, China), yielding a final titer of 1.93 × 10^13^ vector genomes (vg) per mL. PHP.eB‐EGFP virus (Taitool Bioscience, #S0263‐PeB) with a titer of 1 × 10^13^ vg mL^−1^ was also used in this study. For in vivo delivery, P1 WT mice were subjected to hypothermic anesthesia and subsequently administered 1.5 µL of viral suspension via round window membrane (RWM) injection into the left ear using a finely pulled glass micropipette. The contralateral (right) ear served as the non‐injected control. Following the procedure, the surgical site underneath the injected ear was sealed with Vetbond tissue adhesive (3 m, #1469SB), and mice were maintained on a 37 °C warming platform until thermoregulatory recovery was achieved before being returned to maternal care.

### Statistical Analysis

All quantitative data were derived from a minimum of three independent replicates, with sample sizes (n) corresponding to either the number of animals or the experimental repetitions as indicated in the figures or figure legends. Statistical analyses were conducted in GraphPad Prism software (version 10), and the values are expressed as the mean ± SEM (standard error of the mean). Intergroup comparisons were assessed using two‐tailed unpaired Student's *t*‐tests, with statistical significance defined as *p* < 0.05.

### Ethics Approval Statement

All animal experiments were conducted in strict accordance with protocols approved by the Animal Care and Use Committee of Southeast University (experiment number: SEU‐IACUC‐20250902004) following the Guide for the Care and Use of Laboratory Animals outlined by the National Institutes of Health. Every effort was made to minimize animal usage and to alleviate potential distress in the experimental procedures

## Conflict of Interest

The authors declare no conflict of interest.

## Supporting information



Supporting Information

## Data Availability

The data that support the findings of this study are available from the corresponding author upon reasonable request.
