# Supplementary material for: Cidea Targeting Protects Cochlear Hair Cells and Hearing Function From Drug‐ and Noise‐Induced Damage
Source: Adv Sci (Weinh). 2025 Nov 20;13(7):e17206. doi: 10.1002/advs.202517206 (PMC12866833; doi:10.1002/advs.202517206)
Supplement: Supplementary file 1 — Supporting Information [file ADVS-13-e17206-s001.docx]

Supporting Information

**Cidea Targeting Protects Cochlear Hair Cells and Hearing Function from Drug- and Noise-Induced Damage**

Shasha Zhang*, Ruiying Qiang, Yuan Zhang, Jinxian Wan, Chen Tao, Ying Dong, Xujun Tang, Li Xu, Hairong Xiao, Yanqin Lin, Wei Tong, Ying Ma, Yongming Wang*, Peng Li*, Renjie Chai*

**Supplementary materials and methods:**

**Cell Culture and Transfection**

HEI-OC1 cells were cultured in high-glucose DMEM (Gibco, 11995500) supplemented with 10% fetal bovine serum (FBS) (Pansera, P30-2602) at 37°C with 10% CO_2_. Cells were treated with 2 mM neomycin in DMEM without FBS for 24 h and then allowed to recover for another 48 h. Lipofectamine 2000 (Invitrogen, 11668027) and Lipofectamine RNAimax (Invitrogen, 13778075) transfection reagents were used to transfect plasmids and siRNAs into cells according to the manufacturer’s instructions, respectively. The pCMV-HA-Cidea plasmid was a gift from Professor Peng Li of Tsinghua University [1]. Three siRNAs for *Cidea* were designed for knocking down *Cidea*. The sequences of the three siRNAs were as follows: si620: 5’-GAG UCA CCU UCG ACC UAU ATT-3’; si588: 5’-CUG CAA GCA ACC AAA GAA ATT-3’; si683: 5’-CCA CGA UGU ACG AGA UGU ATT-3’.

**Flow Cytometry**

An Annexin V-FITC Apoptosis Detection Kit (C1062, Beyotime) was used to measure the level of apoptosis following the manufacturer’s instructions. After treating the HEI-OC1 cells with neomycin and/or transfecting with plasmids or siRNAs, the cells were trypsinized, washed twice with PBS, and resuspended in binding buffer at a concentration of 5 × 10^5^ cells/ml. Annexin V-FITC and propidium iodide (PI) were added and gently mixed with the cells and incubated for 10 min at room temperature in the dark. Cells were analyzed as quickly as possible by flow cytometry (BD FACS Aria III).

**CRISPR-Cas9 Genome Editing and Target Site Analysis**

The target sequences of three sgRNAs for editing Cidea were as follows (Figure S6A): Cidea-sgRNA1: 5'-GGG CGA GCT GGA TGT ATG AGG GGGG-3'; Cidea-sgRNA2: 5'-AAG GGC GAG CTG GAT GTA TGA GGGG-3'; Cidea-sgRNA3: 5'-CAT GAC CGA AGT AGC CGG CGT GGGG-3'. Five days after transfecting each sgRNA and the SlugCas9-HF-containing plasmids (Figure S6B) into N2a cells, genomic DNA was extracted using QuickExtract DNA Extraction Solution 1.0 (Lucigen, QE09050). The targeted genomic regions were amplified by PCR and subsequently purified with a TIANgel Midi Purification Kit (TIANGEN, DP209-03) to prepare samples for deep sequencing analysis. The following primer pair was used for amplification: forward: 5'-GTC TGA AAA ATG GTT CTT TCC CCT C-3'; reverse: 5'-TCT TAT CAC TGG CTT ACT GCC C-3'.

**Supplementary Figures:**

**
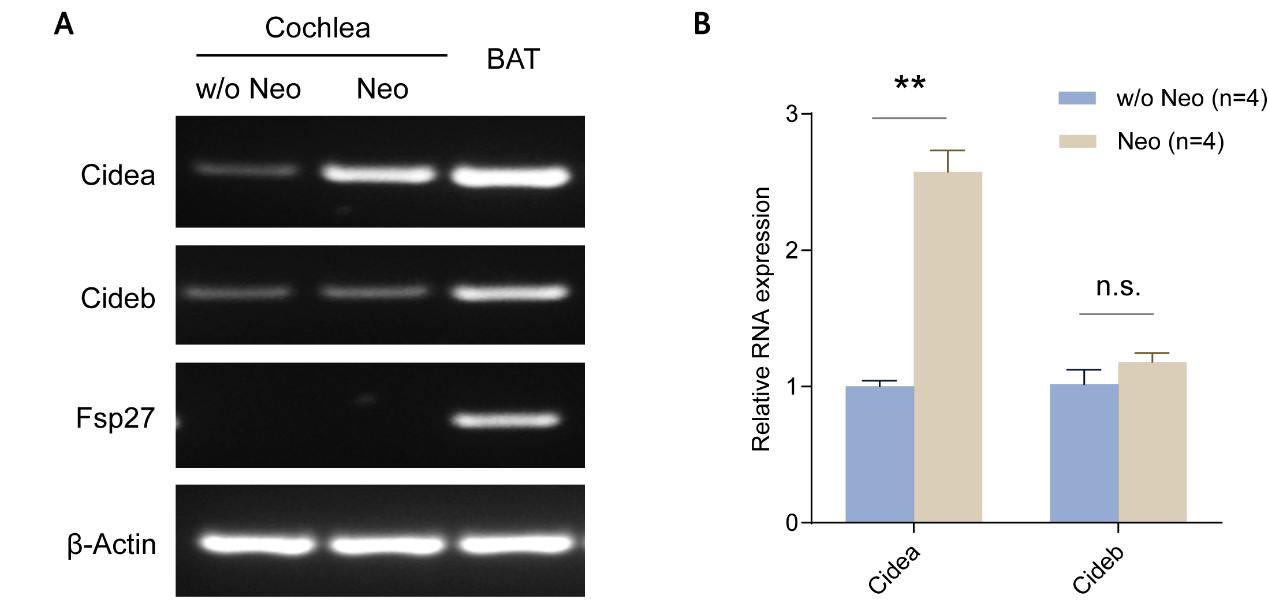
**

**Figure S1.** The expression of Cide family members in the cochlea. **(A, B)** The expression level of three Cide genes (Cidea, Cideb, Fsp27) in the cochlea after neomycin (Neo) damage *ex vivo* was quantified by RT-PCR (A) and RT-qPCR (B), respectively. BAT was used as positive control. w/o, without. n.s., not significant. **, p<0.001.


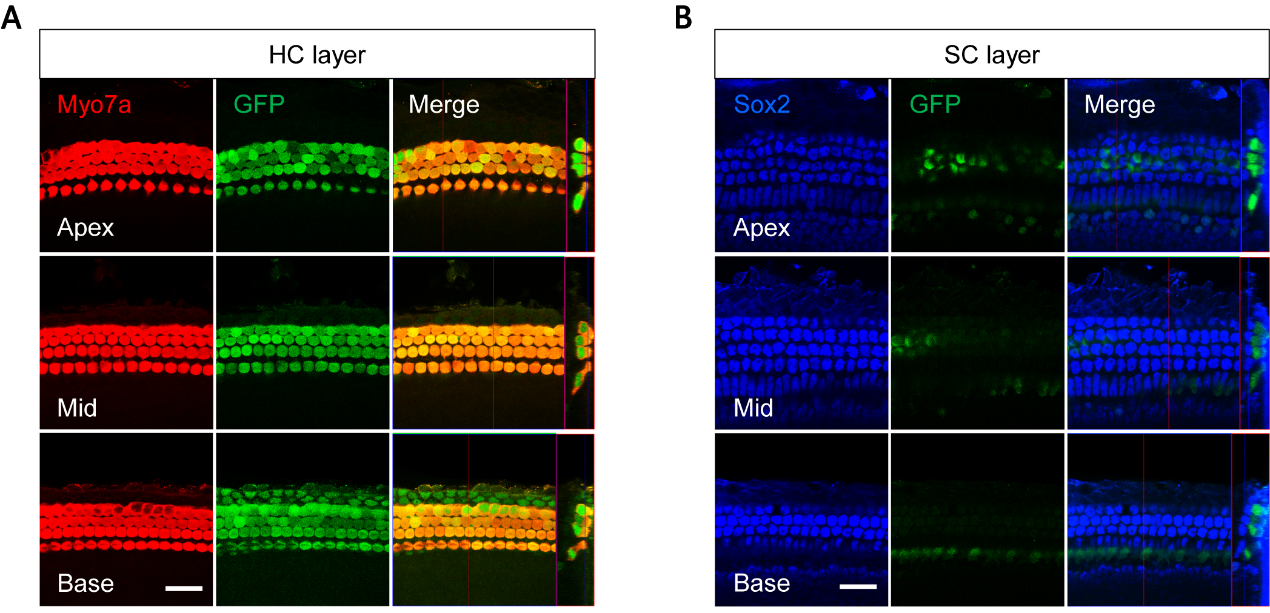


**Figure S2.** Cochlear HCs of Atoh1-GFP mice are GFP+. **(A, B)** P3 Atoh1-GFP mice cochlea was dissected and immunostained. HC layer (A) and SC layer (B) were shown here. Myo7a was used as HC marker, and Sox2 was used as SC marker. Scale bar, 20 μm.


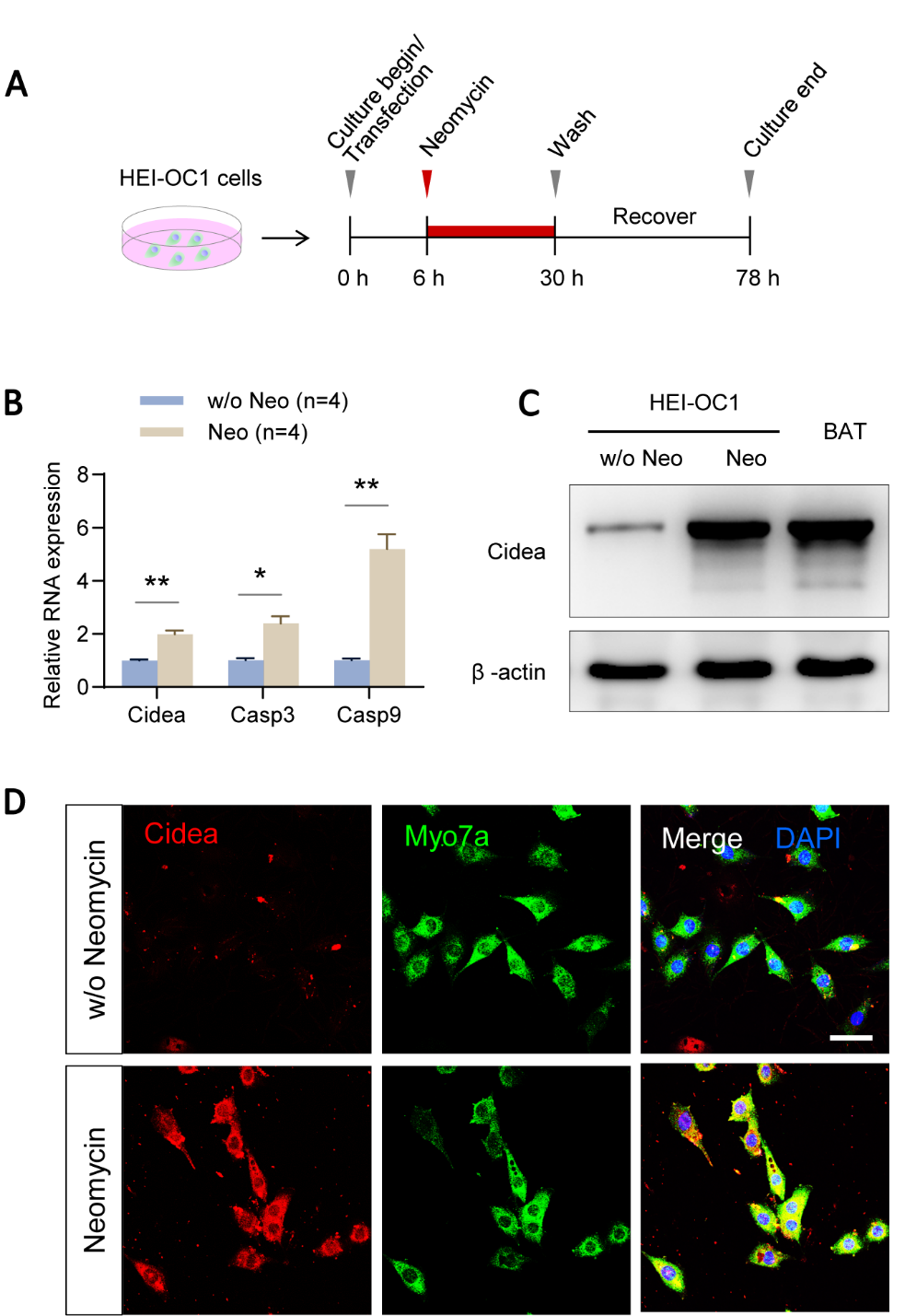


**Figure S3.** Cidea expression is specifically upregulated in neomycin-damaged HEI-OC1 cells. **(A)** The flow chart of the *in vitro* neomycin (Neo) damage model in HEI-OC1 cell line. The cells were treated with 2.0 mM neomycin in DMEM without FBS. **(B-D)** Cidea expression was assessed by RT-qPCR (B), Western blot (C), and immunofluorescent staining (D) in HEI-OC1 cells after 2 mM neomycin treatment. BAT was used as positive control. Scale bar, 50 μm in (D). w/o, without. **, p<0.01. ***, p<0.001.


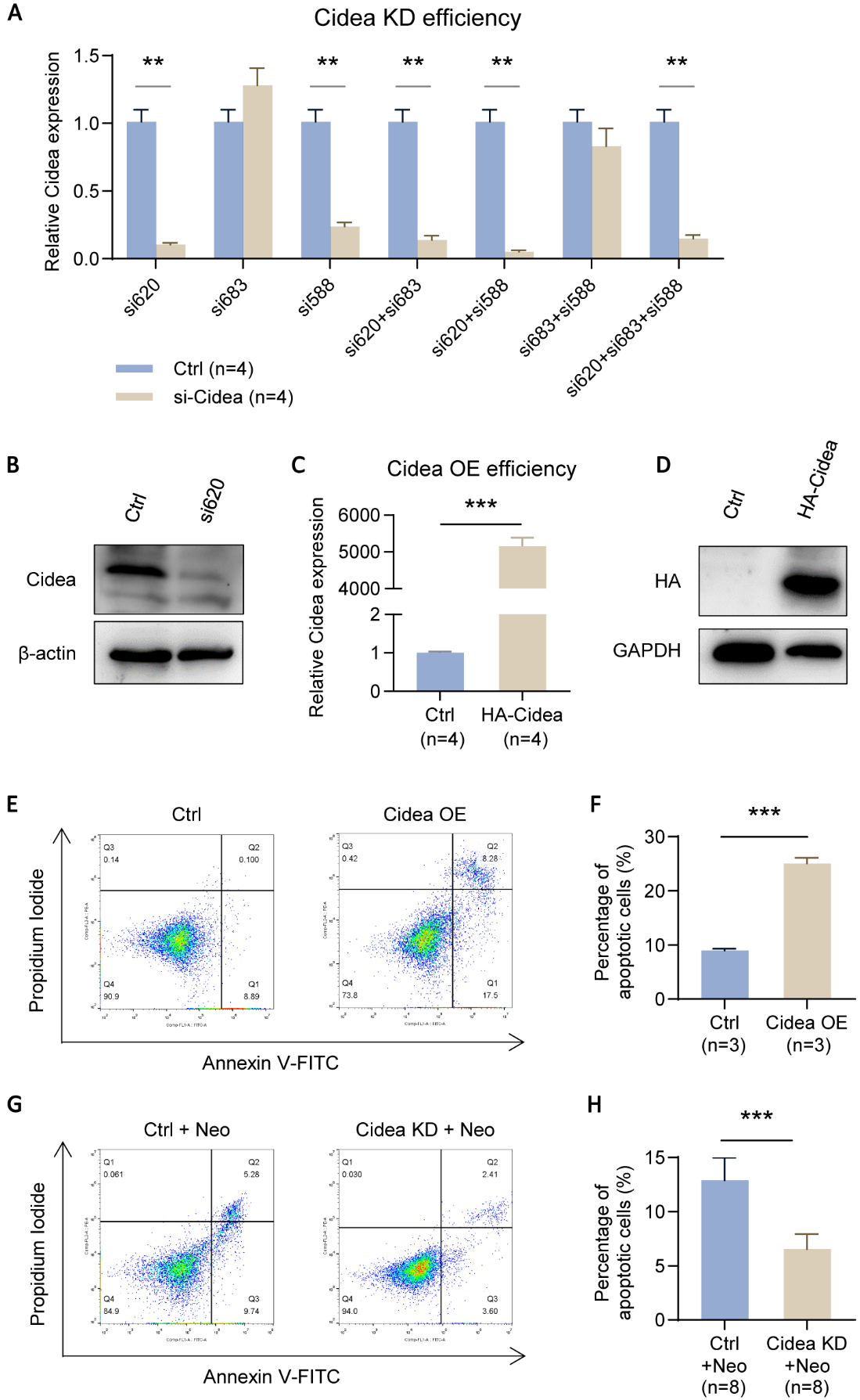


**Figure S4.** Cidea regulate apoptosis in HEI-OC1 cells. **(A, B)** Three siRNAs (si620, si683, si588) were designed for Cidea knockdown in HEI-OC1 cells, and their knockdown efficiency was quantified by RT-qPCR (A) and Western blot (B). The most efficient si620 was used in (B). **(C, D)** pCMV-HA-Cidea plasmids were transfected into HEI-OC1 cells, and the overexpression efficiency of Cidea was assessed by RT-qPCR (C) and western blot (D). **(E-H)** The apoptotic cells were stained using an Annexin V/PI kit and were quantified by FAC sorting after Cidea overexpression (OE) by transfecting HEI-OC1 cells with the pCMV6-HA-Cidea plasmid (E, F) or after Cidea knockdown (KD) by transfecting cells with of Cidea-siRNA and neomycin (Neo) treatment (G, H). **, p<0.01. ***, p<0.001.


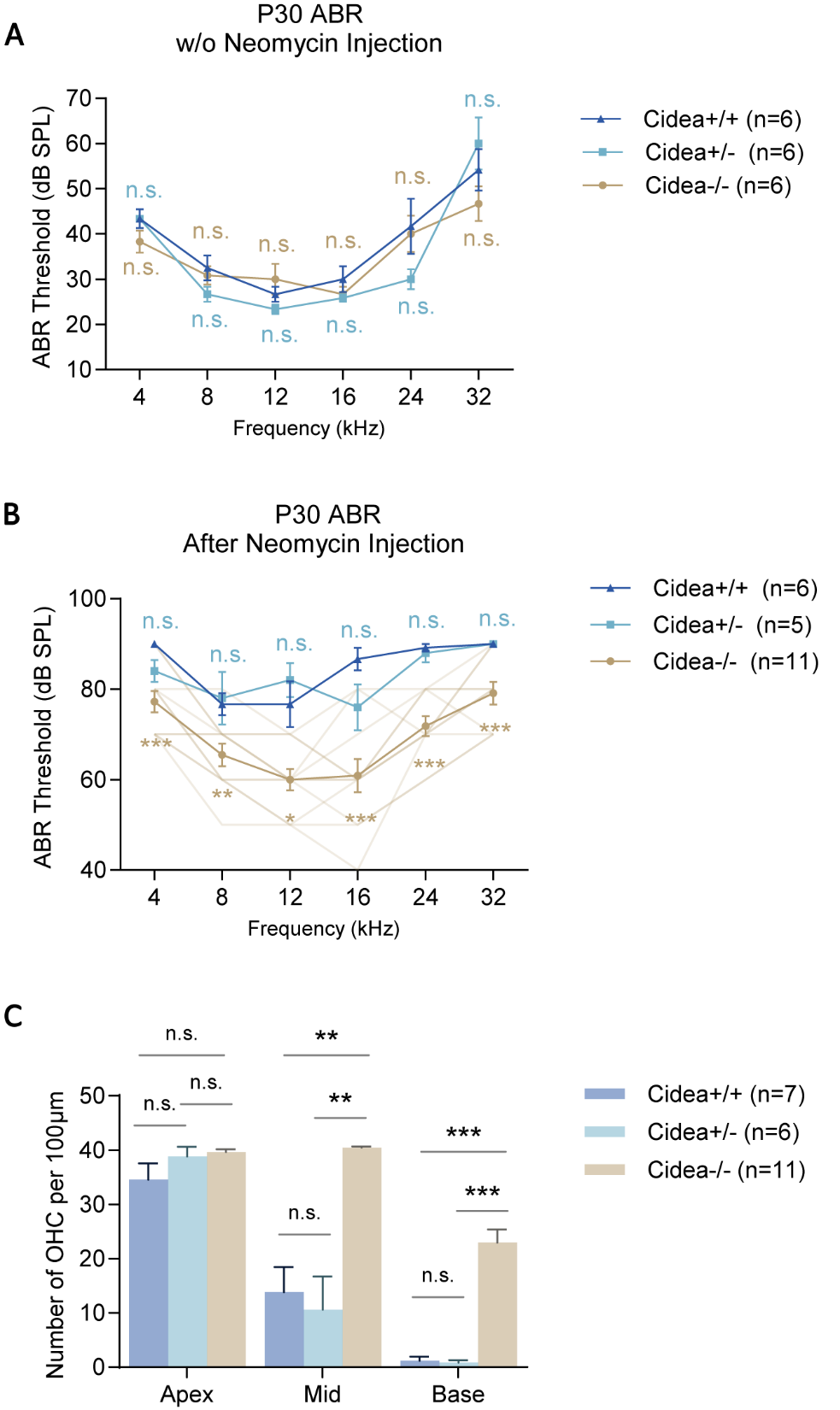


**Figure S5.** ABR test and HC number of Cidea transgenic mice without and after neomycin injection *in vivo*. **(A, B)** ABR hearing threshold shift of P30 WT Cidea+/+ mice, Cidea+/-mice, and Cidea-/- mice was tested without (A) and after (B) neomycin i.p. injection *in vivo*. The green n.s. indicates not significant between WT and Cidea+/- mice. The Yellow n.s. and asterisks indicates not significant and significant between WT and Cidea-/- mice, respectively. The lighter yellow lines in (B) represent the ABR threshold of individual Cidea-/- mice. **(C)** Cochlear OHCs of P30 Cidea+/+ mice, Cidea+/-mice, and Cidea-/- mice were quantified after neomycin i.p. injection *in vivo*. n.s., not significant. **, p<0.01. ***, p<0.001.


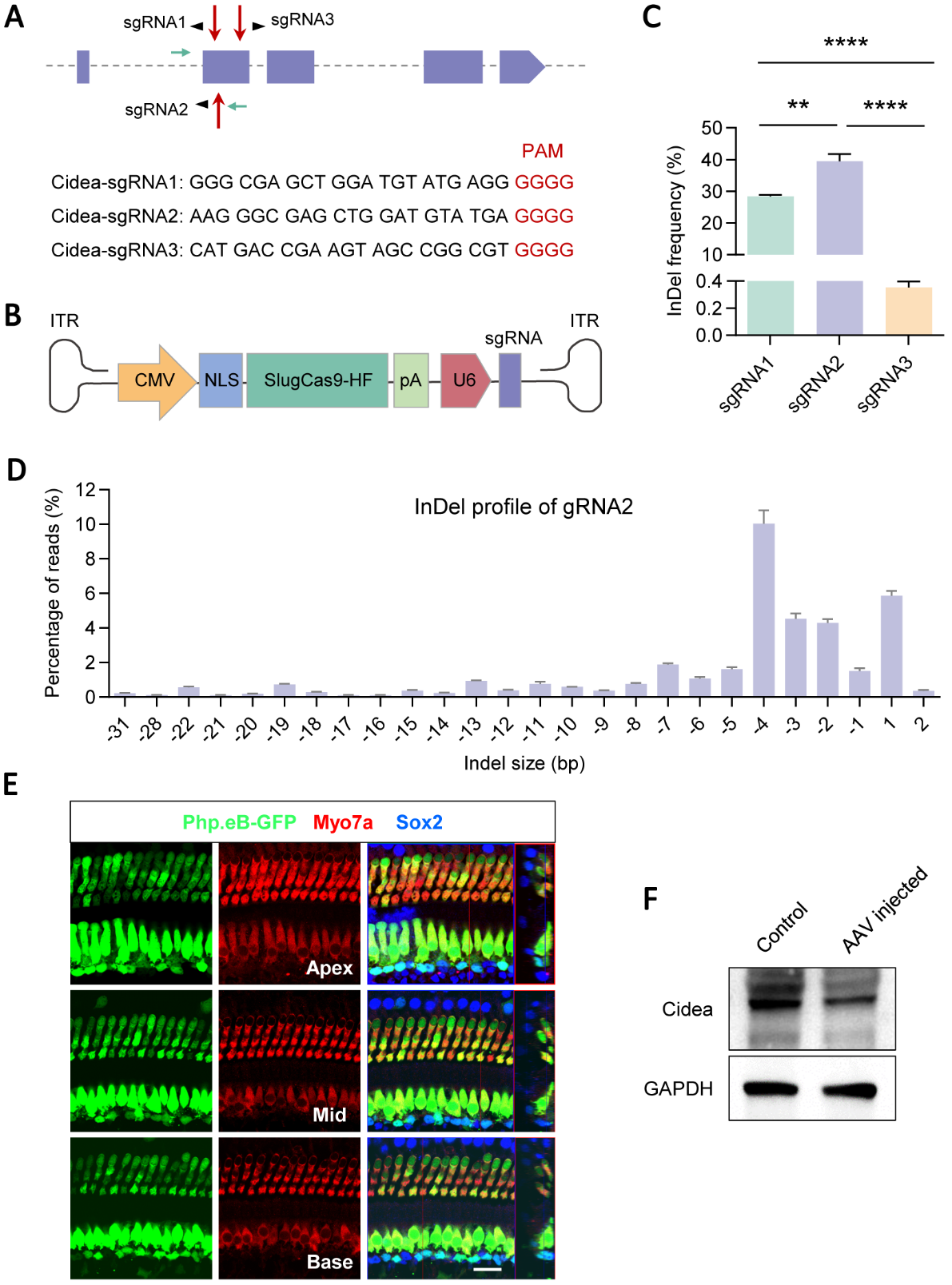


**Figure S6.** PHP.eB-SlugCas9-HF-sgRNA2 could effectively edit the *Cidea* gene in the cochlea. **(A)** The three sgRNAs designed for editing the *Cidea* gene. Red arrows indicate sgRNA target exon sites and the black arrowheads indicate the sgRNA directions. Blue arrows indicate the sequencing primers. The sequences of the sgRNAs are listed, and the PAM sites are marked in red. **(B)** The schematic of the SlugCas9-HF-Cidea-sgRNA2 plasmid. NLS, nuclear localization sequence. pA, poly A. ITR, inverted terminal repeat. **(C)** The indel frequencies of the three sgRNAs were determined by deep sequencing. **(D)** The indel profile of the sgRNA2. Indel frequencies more than 0.1% are shown. Negative and positive numbers for indel size represent deletions and insertions, respectively. n = 3 in each group in (C) and (D). **(E)** A total of 1.5 μL PHP.eB-EGFP virus with a titer of 1 × 10^13^ vg/mL was injected into P1 mice cochleae though the RWM, and the infection efficiency was determined at P7 by immunofluorescent staining. Myo7a was used as the HC marker and Sox2 was used as the SC marker. Scale bar, 20 μm. **(F)** A total of 1.5 μL PHP.eB-SlugCas9-HF-sgRNA2 virus with a titer of 1.93 × 10^13^ vg/mL was injected into the left ear of P1 WT mice though the RWM, and the editing efficiency of *Cidea* was determined at P7 by Western blot. The non-injected ear was used as the control.

**Supplementary Tables:**

**Table S1** RT-qPCR primers

| Primers | Sequences |
| --- | --- |
| Cidea-Forward | 5’-TGACATTCATGGGATTGCAGAC-3’ |
| Cidea-Reverse | 5’- GGCCAGTTGTGATGACTAAGAC-3’ |
| Cideb-Forward | 5’-CAATGGCCTGCTAAGGTCAGT-3’ |
| Cideb-Reverse | 5’-GATCACAGACACGGAAGGGTC-3’ |
| Fsp27-Forward | 5’-ATGGACTACGCCATGAAGTCT-3’ |
| Fsp27-Reverse | 5’-CGGTGCTAACACGACAGGG-3’ |
| Caspase 3-Forward | 5’-CTGACTGGAAAGCCGAAACTC -3’ |
| Caspase 3-Reverse | 5’-CGACCCGTCCTTTGAATTTCT -3’ |
| Caspase 9-Forward | 5’-TCCTGGTACATCGAGACCTTG-3’ |
| Caspase 9-Reverse | 5’- AAGTCCCTTTCGCAGAAACAG-3’ |
| β-actin-Forward | 5’- ACG GCCAGGTCA TCA CTA TTG -3’ |
| β-actin-Reverse | 5’- AGGGGCCGGACT CAT CGT A -3’ |
| GAPDH-Forward | 5’- AGGTCGGTGTGAACGGATTTG -3’ |
| GAPDH-Reverse | 5’-TGTAGACCATGTAGTTGAGGTCA -3’ |

**Table S2** HC counts of neomycin damage model *ex vivo*

|  | w/o Neomycin | |  | Neomycin | |
| --- | --- | --- | --- | --- | --- |
|  | *Cidea+/+*  (n=6) | *Cidea-/-*  (n=6) |  | *Cidea+/+*  (n=6) | *Cidea-/-*  (n=6) |
| HCs per 100 μm (Apex) | 56.20 ± 1.81 | 55.16 ± 2.37 |  | 54.79 ± 1.91 | 55.00 ± 1.84 |
| HCs per 100 μm (Middle) | 51.41 ± 2.01 | 54.17 ± 1.24 |  | 6.30 ± 2.82 | 56.90± 1.43 |
| HCs per 100 μm (Base) | 49.27 ± 3.35 | 53.07 ± 0.75 |  | 6.85 ± 2.56 | 18.62 ± 1.36 |

**Table S3** TUNEL+ cell counts of neomycin damage model *ex vivo*

|  | Neomycin | |
| --- | --- | --- |
|  | *Cidea+/+*  (n=6) | *Cidea-/-*  (n=5) |
| TUNEL+ cells per 100 μm (Apex) | 0.16 ± 0.08 | 0.09 ± 0.06 |
| TUNEL+ cells per 100 μm (Middle) | 16.46 ± 1.39 | 0.84 ± 0.27 |
| TUNEL+ cells per 100 μm (Base) | 20.78 ± 1.89 | 10.13 ± 1.14 |

**Table S4** ABR thresholds of neomycin damage model *in vivo*

|  | w/o Neomycin injection | |  | Neomycin injection | |
| --- | --- | --- | --- | --- | --- |
| Frequency | *Cidea+/+*  (n=6) | *Cidea-/-*  (n=6) |  | *Cidea+/+*  (n=6) | *Cidea-/-*  (n=11) |
| 4 kHz | 43.33 ± 2.11 | 38.33 ± 2.47 |  | 90.00 ± 0.00 | 77.27 ± 2.37 |
| 8 kHz | 32.50 ± 2.81 | 30.83 ± 2.01 |  | 76.67 ± 2.47 | 65.45 ± 2.47 |
| 12 kHz | 26.67 ± 1.67 | 30.00 ± 3.42 |  | 76.67 ± 5.11 | 60.00 ± 2.34 |
| 16 kHz | 30.00 ± 2.89 | 26.67 ± 1.67 |  | 86.67 ± 2.47 | 60.91 ± 3.68 |
| 24 kHz | 41.67 ± 6.15 | 40.00 ± 4.08 |  | 89.17 ± 0.83 | 71.82 ± 2.26 |
| 32 kHz | 51.17 ± 4.55 | 46.67 ± 3.80 |  | 90.00 ± 0.00 | 79.09 ± 2.51 |

**Table S5** OHC counts of neomycin damage model *in vivo*

|  | w/o Neomycin injection | |  | Neomycin injection | | |  |
| --- | --- | --- | --- | --- | --- | --- | --- |
|  | *Cidea+/+*  (n=4) | *Cidea-/-*  (n=4) |  | *Cidea+/+*  (n=7) | *Cidea+/-*  (n=6) | *Cidea-/-*  (n=12) |  |
| OHCs per 100 μm (Apex) | | 39.77 ± 0.27 | 39.92 ± 0.23 |  | 34.53 ± 3.05 | 38.85 ± 1.76 | 39.62 ± 0.55 |
| OHCs per 100 μm (Middle) | | 39.06 ± 0.22 | 39.45 ± 0.27 |  | 13.82 ± 4.60 | 10.57± 6.14 | 40.45 ± 0.21 |
| OHCs per 100 μm (Base) | | 38.59 ± 0.20 | 38.59 ± 0.20 |  | 1.21 ± 0.74 | 0.86 ± 0.46 | 22.93 ± 2.45 |

**Table S6** ABR thresholds of noise damage model *in vivo*

|  | before Noise exposure | |  | after Noise exposure | |
| --- | --- | --- | --- | --- | --- |
| Frequency | *Cidea+/+*  (n=10) | *Cidea-/-*  (n=11) |  | *Cidea+/+*  (n=10) | *Cidea-/-*  (n=11) |
| 4 kHz | 49.5 ± 2.83 | 51.36 ± 3.10 |  | 85.00 ± 2.24 | 72.72 ± 1.95 |
| 8 kHz | 29.00 ± 1.80 | 31.82 ± 2.72 |  | 85.00 ± 3.42 | 64.09 ± 3.08 |
| 12 kHz | 23.00 ± 2.13 | 29.09 ± 6.53 |  | 80.00 ± 3.33 | 61.82 ± 2.96 |
| 16 kHz | 27.00 ± 2.38 | 23.64 ± 2.25 |  | 82.00 ± 2.91 | 55.00 ± 2.43 |
| 24 kHz | 30.00 ± 2.24 | 31.82 ± 2.80 |  | 84.00 ± 3.06 | 68.64 ± 3.02 |
| 32 kHz | 36.50 ± 1.67 | 39.09 ± 3.22 |  | 86.00 ± 2.67 | 77.27 ± 2.73 |

**Table S7** OHC counts of noise damage model *in vivo*

|  | after Noise exposure | |  | after Noise exposure | |
| --- | --- | --- | --- | --- | --- |
|  | *Cidea+/+*  (n=4) | *Cidea-/-*  (n=4) |  | *Cidea+/+*  (n=5) | *Cidea-/-*  (n=5) |
| OHCs per 100 μm (Apex) | 39.30 ± 0.89 | 39.30 ± 0.59 |  | 39.53 ± 0.19 | 39.81 ± 0.31 |
| OHCs per 100 μm (Middle) | 39.16 ± 0.44 | 39.94 ± 0.79 |  | 37.50 ± 1.11 | 39.44 ± 0.28 |
| OHCs per 100 μm (Base) | 37.43 ± 0.40 | 38.72 ± 0.65 |  | 11.28 ± 6.82 | 35.69 ± 0.51 |

**Table S8** ABR thresholds of neomycin damage model after AAV injection *in vivo*

|  | after Neomycin injection | |
| --- | --- | --- |
| Frequency | AAV non-injected ears  (n=9) | AAV injected ears  (n=9) |
| 4 kHz | 66.67 ± 5.14 | 53.89 ± 2.73 |
| 8 kHz | 58.89 ± 6.55 | 39.44 ± 2.42 |
| 12 kHz | 70.00 ± 4.71 | 51.11 ± 5.32 |
| 16 kHz | 78.89 ± 2.17 | 63.33 ± 3.82 |
| 24 kHz | 81.11 ± 1.82 | 73.89 ± 1.11 |
| 32 kHz | 83.33 ± 2.20 | 76.11 ± 2.17 |

**Table S9** HC counts of neomycin damage model after AAV injection *in vivo*

|  | after Neomycin injection | |
| --- | --- | --- |
|  | AAV non-injected ears  (n=9) | AAV injected ears  (n=9) |
| OHCs per 100 μm (Apex) | 38.89 ± 0.84 | 39.44 ± 0.50 |
| OHCs per 100 μm (Middle) | 18.33 ± 5.62 | 35.56 ± 2.81 |
| OHCs per 100 μm (Base) | 0.22 ± 0.22 | 4.44 ± 1.61 |

**Table S10** ABR thresholds of noise damage model after AAV injection in vivo

|  | after Noise exposure | |
| --- | --- | --- |
| Frequency | AAV non-injected ears  (n=12) | AAV injected ears  (n=12) |
| 4 kHz | 90.00 ± 0.00 | 72.92 ± 4.15 |
| 8 kHz | 81.25 ± 3.54 | 63.33 ± 5.45 |
| 12 kHz | 72.08 ± 5.31 | 56.25 ± 4.89 |
| 16 kHz | 73.33 ± 5.38 | 65.00 ± 4.85 |
| 24 kHz | 77.92 ± 3.96 | 68.33 ± 4.97 |
| 32k Hz | 81.67 ± 3.50 | 68.75 ± 4.13 |

**Table S11** HC counts of noise damage model after AAV injection *in vivo*

|  | after Noise exposure | |
| --- | --- | --- |
|  | AAV non-injected ears  (n=12) | AAV injected ears  (n=12) |
| OHCs per 100 μm (Apex) | 37.75 ± 0.57 | 38.58 ± 0.50 |
| OHCs per 100 μm (Middle) | 36.83 ± 1.80 | 38.83 ± 0.81 |
| OHCs per 100 μm (Base) | 26.00 ± 1.84 | 33.08 ± 1.73 |

**Reference:**

1. Wang, W.S., et al., *Cidea is an essential transcriptional coactivator regulating mammary gland secretion of milk lipids.* Nature Medicine, 2012. **18**(2): p. 235-243.
